# Supplementary figures and images for: Predicting patients with false negative SARS-CoV-2 testing at hospital admission: A retrospective multi-center study
Source: PLoS One. 2021 May 12;16(5):e0251376. doi: 10.1371/journal.pone.0251376 (PMC8115794; doi:10.1371/journal.pone.0251376)

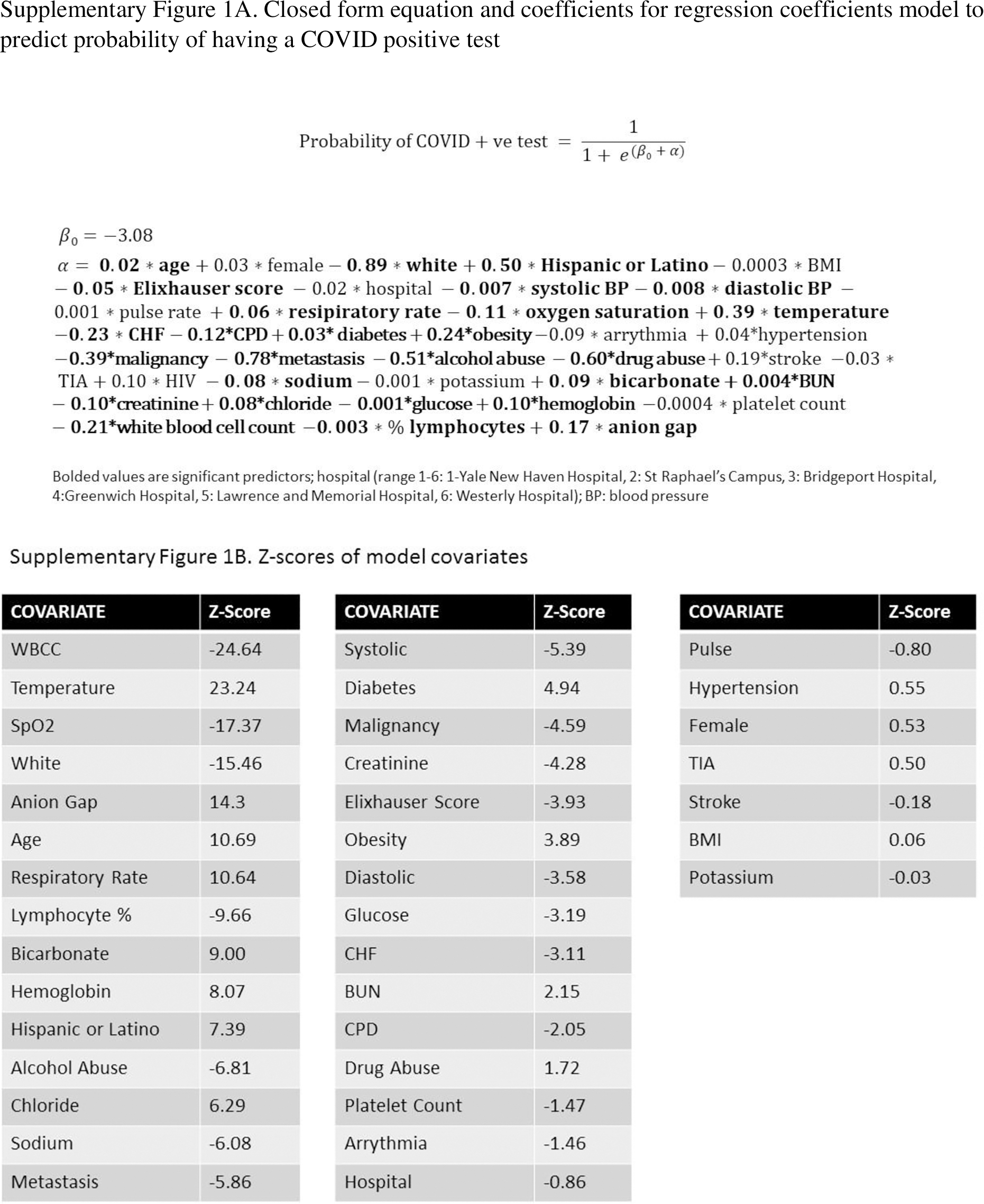

Supplement: S1 Fig — A. Closed form equation and coefficients for regression coefficients model to predict probability of having a COVID positive test. Bolded values are significant predictors; hospital (range 1–6: 1-Yale New Haven Hospital, 2: St Raphael’s Campus, 3: Bridgeport Hospital, 4: Greenwich Hospital, 5: Lawrence and Memorial Hospital, 6: Westerly Hospital); BP: blood pressure. B. Z-scores of model covariates. (TIF) [file pone.0251376.s001.tif]

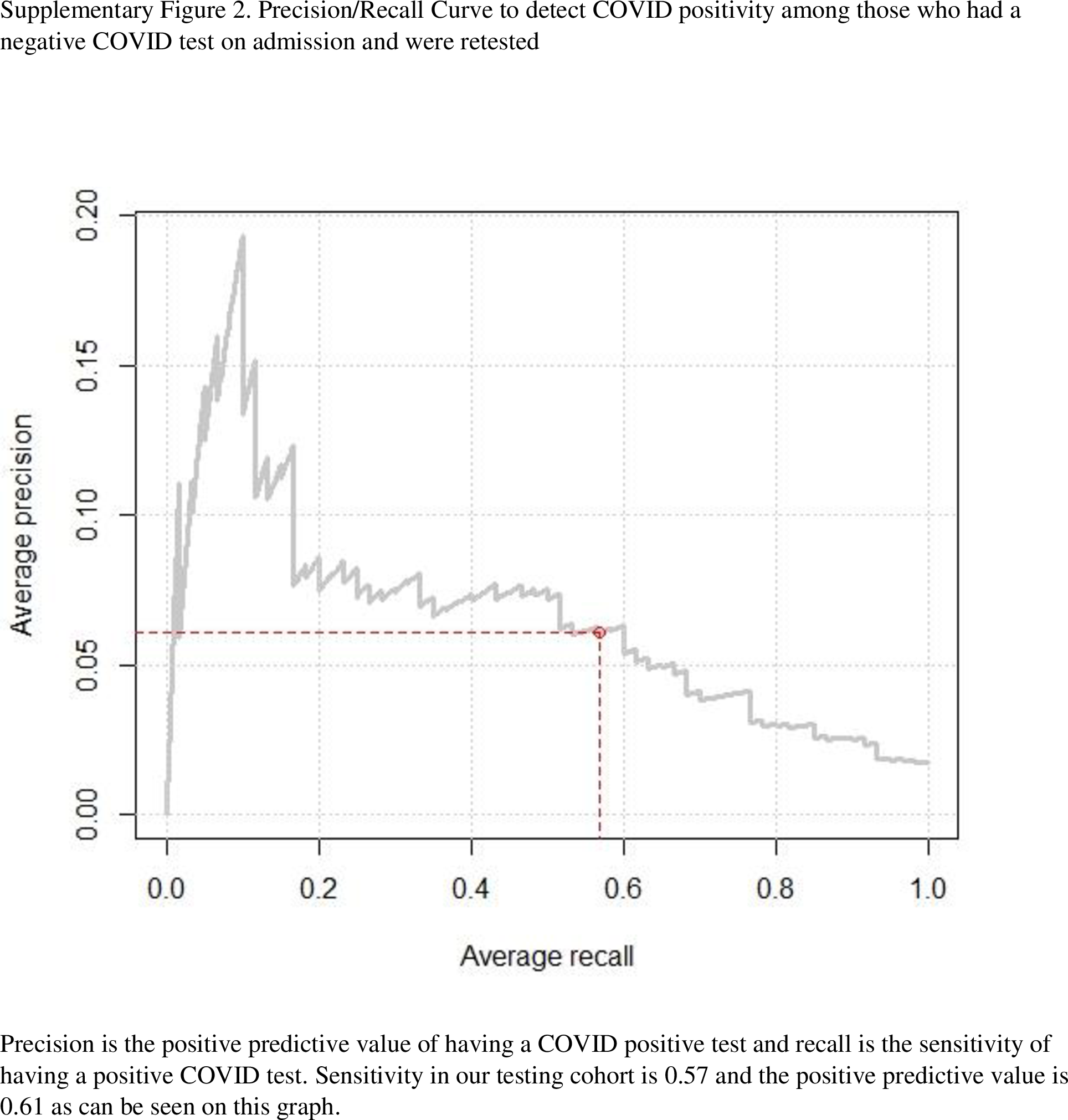

Supplement: S2 Fig — Precision is the positive predictive value of having a COVID positive test and recall is the sensitivity of having a positive COVID test. Sensitivity in our testing cohort is 0.57 and the positive predictive value is 0.61 as can be seen on this graph. (TIF) [file pone.0251376.s002.tif]
