# Supplementary material for: Predicting patients with false negative SARS-CoV-2 testing at hospital admission: A retrospective multi-center study
Source: PLoS One. 2021 May 12;16(5):e0251376. doi: 10.1371/journal.pone.0251376 (PMC8115794; doi:10.1371/journal.pone.0251376)
Supplement: Attachment — Submitted filename: Response to Reviewers 2021_04_22.docx [file pone.0251376.s004.docx]

EPONE-D-20-38011
Predicting patients with false negative SARS-CoV-2 testing at hospital admission: A retrospective multi-center study

**Response to Reviewers**

*Editorial Comments*

*1) Please review your reference list to ensure that it is complete and correct. If you have cited papers that have been retracted, please include the rationale for doing so in the manuscript text, or remove these references and replace them with relevant current references. Any changes to the reference list should be mentioned in the rebuttal letter that accompanies your revised manuscript. If you need to cite a retracted article, indicate the article’s retracted status in the References list and also include a citation and full reference for the retraction notice.*

**Author’s Reply:** We have reviewed the reference list to ensure completeness and accuracy.

*2) Please ensure that your manuscript meets PLOS ONE's style requirements, including those for file naming. The PLOS ONE style templates can be found at*

[*https://journals.plos.org/plosone/s/file?id=wjVg/PLOSOne_formatting_sample_main_body.pdf*](https://journals.plos.org/plosone/s/file?id=wjVg/PLOSOne_formatting_sample_main_body.pdf) *and*

[*https://journals.plos.org/plosone/s/file?id=ba62/PLOSOne_formatting_sample_title_authors_affiliations.pdf*](about:blank)

**Author’s Reply:** We have reviewed the PLOS ONE style requirements including those for file naming and have made modifications where indicated.

1) *In your Methods section, please provide additional information about the methodology used (the equations representing the model; how the model was calibrated; and what parameters were applied).*

**Author’s Reply**: Thank you for this comment. We include the below text in the manuscript to provide information regarding the methodology:

We trained a logistic regression model to predict COVID-positivity in patients with an initial positive COVID test (+/0) and those with an initial negative COVID test who were never retested (-/0). We then tested the performance of this model amongst individuals with an initial negative COVID test who were retested and negative (-/-) and retested and positive (-/+) within the first 96 hours of their hospitalization.

…

All analyses were performed using R (Version 4.0.0, Vienna, Austria).^13^ Logistic regression models were developed using the *glm* function from the ‘stats’ package in R. We defined statistical significance at P<0.05.

…

Patient variables were chosen pragmatically for those that would be simpler to embed into a clinical decision support platform either directly onto the EHR or as a web service. These variables were chosen as they contained very low (<10%) missingness for hospitalized patients within the first 24 hours of hospitalization.

*4) Thank you for stating the following in the Funding/Support Section of your manuscript:*

*[**R01DK113191 and P30DK079310 to FPW]*

*We note that you have provided funding information that is not currently declared in your Funding Statement. However, funding information should not appear in the Acknowledgments section or other areas of your manuscript. We will only publish funding information present in the Funding Statement section of the online submission form.*

*Please remove any funding-related text from the manuscript and let us know how you would like to update your Funding Statement. Currently, your Funding Statement reads as follows:*

*[The author(s) received no specific funding for this work.]*

*Please include your amended statements within your cover letter; we will change the online submission form on your behalf.*

**Author’s Reply:** Thank you for this message; we have removed the funding information from the manuscript and have placed a revised funding statement within the cover letter.

*Reviewers' comments:

Reviewer's Responses to Questions*

*Comments to the Author

1. Is the manuscript technically sound, and do the data support the conclusions?

The manuscript must describe a technically sound piece of scientific research with data that supports the conclusions. Experiments must have been conducted rigorously, with appropriate controls, replication, and sample sizes. The conclusions must be drawn appropriately based on the data presented.*

*Reviewer #1: Partly*

*2. Has the statistical analysis been performed appropriately and rigorously?*

*Reviewer #1: Yes*

*3. Have the authors made all data underlying the findings in their manuscript fully available?

The* [*PLOS Data policy*](https://nam12.safelinks.protection.outlook.com/?url=http%3A%2F%2Fwww.plosone.org%2Fstatic%2Fpolicies.action%23sharing&data=04%7C01%7Cmichael.simonov%40yale.edu%7C9c195b0b19984629da3408d8f8eddbf8%7Cdd8cbebb21394df8b4114e3e87abeb5c%7C0%7C0%7C637533047764629716%7CUnknown%7CTWFpbGZsb3d8eyJWIjoiMC4wLjAwMDAiLCJQIjoiV2luMzIiLCJBTiI6Ik1haWwiLCJXVCI6Mn0%3D%7C1000&sdata=mFg3umUb6L629HprWxophm6J%2F3WqIIl2H%2BgaDgAd7AI%3D&reserved=0) *requires authors to make all data underlying the findings described in their manuscript fully available without restriction, with rare exception (please refer to the Data Availability Statement in the manuscript PDF file). The data should be provided as part of the manuscript or its supporting information, or deposited to a public repository. For example, in addition to summary statistics, the data points behind means, medians and variance measures should be available. If there are restrictions on publicly sharing data—e.g. participant privacy or use of data from a third party—those must be specified.*

*Reviewer #1: Yes*

*4. Is the manuscript presented in an intelligible fashion and written in standard English?

PLOS ONE does not copyedit accepted manuscripts, so the language in submitted articles must be clear, correct, and unambiguous. Any typographical or grammatical errors should be corrected at revision, so please note any specific errors here.*

*Reviewer #1: Yes*

*5. Review Comments to the Author

Please use the space provided to explain your answers to the questions above. You may also include additional comments for the author, including concerns about dual publication, research ethics, or publication ethics. (Please upload your review as an attachment if it exceeds 20,000 characters)*

*Reviewer #1: The study has very interesting premises aiming to find a simple system to identify false negative COVID-19 patients. However, there are some issues that in my opinion, should be addressed. As a matter of fact, intermittent positive PCR tests for SARS-CoV-2 is a common occurrence but the viability of the virus in those cases is still controversial. The persistence of SARS-CoV-2 and the development of intermittent positive tests may merely indicate the detection of viral RNA. The prolonged shedding of the virus is not an indication of continued infection.
For this reasons, a more accurate evaluation of the false negative population in the study should be provided otherwise the premises for the whole subsequent analysis fail. In this perspective, authors should add some valuable information (at least for the 61 (+/-) patients) like the days from the onset of symptoms to the swab, the reason for hospitalization, SARS-CoV-2 serology, and the immunological status of the included patients (an immunocompromised patient prolonged viral shedding). Would be interesting also understand the reasons for re-testing and the allocation of those patients in the time within the re- test (covid- or no covid ward).*

**Author’s Reply:** Thank you very much for this feedback; we performed a manual validation of the 61 patients who had a negative test followed by positive test to determine these various factors. We have modified the results section of the manuscript accordingly to represent these data:

Manual chart inspection was performed for the 61 patients who retested positive; reasons for subsequent test included high clinical suspicion despite negative test (51%), testing as part of disposition planning (5%) or prior to undergoing a procedure (7%), testing prior to hospital transfer (3%), inconclusive first COVID test (2%), as well as unclear reason for testing (31%). Clinical suspicion included a wide variety of symptoms and findings including abnormal imaging, new-onset fever, hypoxia, shortness of breath, and known contact with a patient with COVID-19. 40% of patients who retested positive did not have symptoms on admission. The mean number of days between first and second test was 2.5 days (IQR: 1 – 2 days).

*Finally in the methods authors should include all types of RT-PCR kits, regardless and the brand or manufacturer, the extraction method used, the number of target gene assays assessed, or the cycle threshold value 136 for positivity.*

**Author’s Reply:** Thank you for this feedback, we have included the types of testing performed across out institution as per our Virology laboratory and have included this in the manuscript methods section:

SARS-CoV-2 tests included several multiplex real time RT-PCR tests (GeneXpert – Cepheid; Siplexa – Diasorin; TaqPath – Thermo Fisher), transcription mediated amplification test (Panther - Hologic) and a singleplex real time RT PCR test (CDC – lab developed). Data regarding specific test used for each sample were not available for this study.

*Minor issues:

Authors in the introduction should clearly state which test are they talking about.*

**Author’s Reply:** Thank you for this comment; we have modified the introduction as follows:

In this retrospective study, we evaluate this group of patients who initially test COVID negative per nasopharyngeal polymerase chain reaction (PCR) testing but subsequently retest positive to identify patient characteristics, vital signs, and laboratory tests that may predict a subsequent positive test for COVID.

*“… approximately 30% of test in COVID patients are false negative…”. It looks a very high percentage. This data is controversial. Several studies report a lower rate. Authors should explain that and mitigate this affirmation.*

**Author’s Reply:** Thank you for this feedback. We have revised the statement using more recent data from a systematic review from this journal which we believe demonstrates a more accurate representation of the false negative rates.

Though COVID tests used in hospital settings are very specific, sensitivity is much lower with significant temporal variability of viral shedding; moreover, a recent systematic review reports a false negative rate of 13%, a number sufficiently high to be clinically meaningful.

*Please provide P value in the table with same number of decimals*

**Author’s Reply:** Thank you for this feedback; we have modified the table accordingly such that the p-values are reported with only one significant digit.

*In my opinion a table in the main text that clearly indicates how to identify the patients who are most likely to be positive in a re-test is missing. The table would help the reader and add clear practical value to the work.*

**Author’s Reply:** Thank you very much for this feedback. We have modified the text to provide a description of how the model could be deployed and how patients could be identified who meet ‘reasonable’ risk of having COVID:

For ready deployment of the model, institutions may apply the model formula presented in **Supplementary Figure 1A** and selecting a cutpoint that aligns with the goals and testing capabilities of the institution (as per above we highlight a cutpoint at the 90^th^ percentile).

In addition, we have modified Supplementary Figure 1B (shown below) for the variables to be in descending order of the absolute value of the z-score. This aids in interpretation of a patient’s risk factors; namely one can see that lower white blood cell count, higher temperature, lower oxygen saturation, and non-white race are the most predictive variables that may guide clinical suspicion.

Supplemental Figure 1B. Z-scores of model covariates

*“……We argue that the model is thus broadly generalizable”… In my opinion authors should specify that this model could be generalizable only for hospitalized patients.*

**Author’s Reply:** Thank you for this comment, we have modified the statement to state that the model is generalizable to hospitalized patients, as per below:

We argue that the model is thus broadly generalizable for hospitalized patients.

*“….To our knowledge, this is the first study to investigate the population of false negative patients with
COVID in the hospitalized setting..” . Indeed, there are some studies analysing false positive patients proposing algorithm to identify them (es. Laboratory markers included in the Corona Score can identify false negative results on COVID-19 RT-PCR in the emergency room. Roberto Assandri et al.). I would re-phrase maybe emphasising that you are not studying false negative, but you are proposing a new model*

**Author’s Reply:** Thank you for this comment; we have removed this text from the manuscript as there are other investigations evaluating false negative testing.

*6. PLOS authors have the option to publish the peer review history of their article (*[*what does this mean?*](https://nam12.safelinks.protection.outlook.com/?url=https%3A%2F%2Fjournals.plos.org%2Fplosone%2Fs%2Feditorial-and-peer-review-process%23loc-peer-review-history&data=04%7C01%7Cmichael.simonov%40yale.edu%7C9c195b0b19984629da3408d8f8eddbf8%7Cdd8cbebb21394df8b4114e3e87abeb5c%7C0%7C0%7C637533047764639707%7CUnknown%7CTWFpbGZsb3d8eyJWIjoiMC4wLjAwMDAiLCJQIjoiV2luMzIiLCJBTiI6Ik1haWwiLCJXVCI6Mn0%3D%7C1000&sdata=mwoPPoK8vzcTshWKAv21dCgpXD%2BJrDafueGgBjXcrrc%3D&reserved=0)*). If published, this will include your full peer review and any attached files.


If you choose “no”, your identity will remain anonymous but your review may still be made public.


Do you want your identity to be public for this peer review? For information about this choice, including consent withdrawal, please see our* [*Privacy Policy*](https://nam12.safelinks.protection.outlook.com/?url=https%3A%2F%2Fwww.plos.org%2Fprivacy-policy&data=04%7C01%7Cmichael.simonov%40yale.edu%7C9c195b0b19984629da3408d8f8eddbf8%7Cdd8cbebb21394df8b4114e3e87abeb5c%7C0%7C0%7C637533047764639707%7CUnknown%7CTWFpbGZsb3d8eyJWIjoiMC4wLjAwMDAiLCJQIjoiV2luMzIiLCJBTiI6Ik1haWwiLCJXVCI6Mn0%3D%7C1000&sdata=FDXZfgpujYPdYuBdMosj%2FjzTSLzEsLQY4E5b31EXgWs%3D&reserved=0)*.*

*Reviewer #1: No*

*[NOTE: If reviewer comments were submitted as an attachment file, they will be attached to this email and accessible via the submission site. Please log into your account, locate the manuscript record, and check for the action link "View Attachments". If this link does not appear, there are no attachment files.]

While revising your submission, please upload your figure files to the Preflight Analysis and Conversion Engine (PACE) digital diagnostic tool,* [*https://pacev2.apexcovantage.com/*](https://nam12.safelinks.protection.outlook.com/?url=https%3A%2F%2Fpacev2.apexcovantage.com%2F&data=04%7C01%7Cmichael.simonov%40yale.edu%7C9c195b0b19984629da3408d8f8eddbf8%7Cdd8cbebb21394df8b4114e3e87abeb5c%7C0%7C0%7C637533047764649708%7CUnknown%7CTWFpbGZsb3d8eyJWIjoiMC4wLjAwMDAiLCJQIjoiV2luMzIiLCJBTiI6Ik1haWwiLCJXVCI6Mn0%3D%7C1000&sdata=5Y7V%2FKodn7Ca8O1y2lTOietAAHcD5je5nThL64iMZ7Y%3D&reserved=0)*. PACE helps ensure that figures meet PLOS requirements. To use PACE, you must first register as a user. Registration is free. Then, login and navigate to the UPLOAD tab, where you will find detailed instructions on how to use the tool. If you encounter any issues or have any questions when using PACE, please email PLOS at* [*figures@plos.org*](mailto:figures@plos.org)*. Please note that Supporting Information files do not need this step.*
